# Dynamics of Rex3 in the genomes of endangered Iberian Leuciscinae (Teleostei, Cyprinidae) and their natural hybrids

**DOI:** 10.1186/s13039-015-0180-1

**Published:** 2015-10-26

**Authors:** Carla Sofia A. Pereira, Marlon F. Pazian, Petr Ráb, Maria João Collares-Pereira

**Affiliations:** Faculty of Sciences, Centre for Ecology, Evolution and Environmental Changes, University of Lisbon, Campo Grande, 1749-016 Lisbon, Portugal; Institute of Animal Physiology and Genetics, Academy of Sciences of Czech Republic, Liběchov, Czech Republic

**Keywords:** *Anaecypris hispanica*, *Chondrostoma s.l.* sp, C_0_t-1 DNA, Karyotype differentiation, Fish hybrids, *Squalius pyrenaicus*, Transposable elements

## Abstract

**Background:**

Iberian Leuciscinae are greatly diverse comprising taxa of hybrid origin. With highly conservative karyotypes, Iberian *Chondrostoma s.l.* have recently demonstrated sub-chromosomal differentiation and rapid genome restructuring in natural hybrids, which was confirmed by ribosomal DNA (rDNA) transposition and/or multiplication. To understand the role of repetitive DNAs in the differentiation of their genomes, a genetic and molecular cytogenetic survey was conducted in *Achondrostoma oligolepis*, *Anaecypris hispanica*, *Iberochondrostoma lemmingii*, *I. lusitanicum*, *Pseudochondrostoma duriense*, *P. polylepis*, *Squalius pyrenaicus* and hybrids between *A. oligolepis* x (*P. duriense*/*P. polylepis*), representing ‘alburnine’, chondrostomine and *Squalius* lineages.

**Results:**

Partial Rex3 sequences evidenced high sequence homology among Leuciscinae (≥98 %) and different fish families (80–95 %) proposing a relatively recent activity of these elements in the species inspected. Low nucleotide substitution rates (<20 %) and intact ORFs suggests that Rex3 may in fact be active in these genomes. The chromosomal distribution of Rex3 retroelement was found highly concentrated at pericentromeric and moderately at subtelomeric blocks, co-localizing with 5S rDNA loci, and correlating with blocks of heterochromatin and C_0_t-1 DNA. This accumulation was evident in at least 10 chromosome pairs, a pattern that seemed to be shared among the different species, likely pre-dating their divergence. Nevertheless, species-specific clusters were detected in *I. lusitanicum*, *P. duriense*, *P. polylepis* and *S. pyrenaicus* demonstrating rapid and independent differentiation. Natural hybrids followed the same patterns of accumulation and association with repetitive sequences. An increased number of Rex3 clusters now associating also with translocated 45S rDNA clusters vouched for other genomic rearrangements in hybrids. Rex3 sequence phylogeny did not agree with its hosts’ phylogeny but the observed distribution pattern is congruent with an evolutionary tendency to protect its activity, a robust regulatory system and/or events of horizontal transfer.

**Conclusions:**

This is the first report directed at retroelement physical mapping in Cyprinidae. It helped outlining conceivable ancestral homologies and recognizing retrotransposon activation in hybrids, being possibly associated with genome diversification within the subfamily. The extensive diversity of Iberian Leuciscinae makes them excellent candidates to explore the processes and mechanisms behind the great plasticity distinguishing vertebrate genomes.

**Electronic supplementary material:**

The online version of this article (doi:10.1186/s13039-015-0180-1) contains supplementary material, which is available to authorized users.

## Background

The subfamily Leuciscinae (Cyprinidae) represents a significant part of the South-European ichthyofauna. High biodiversity and an intricate systematics (reviewed in [1]) make leuciscines very attractive for the investigation of life history, biogeography and speciation within the family (see e.g., [2]). In the Iberian Peninsula, Leuciscinae comprise at least 24 species and cases of extensive natural hybridization encompassing both homoploid and polyploid systems (e.g., [3–5]).

Leuciscinae karyotypes exhibit quite conservative patterns of diploid chromosome numbers (most species have 2n = 50), chromosome categories and few chromosome markers (e.g., [6–8] and references therein). However, the introduction of molecular cytogenetic procedures has demonstrated that such uniformity remains restricted to the level of chromosome macrostructure [9, 10]. Genomes of homoploid hybrids within Iberian *Chondrostoma s.l.* are apparently characterized by rapid genetic restructuring often associated with inter-specific hybridization [11] where transposable elements may play an important role (e.g., [12–15]). Retrotransposons of the Rex family are widely spread among teleost genomes [16–18]. Rex elements were first described in the live-bearing fish *Xiphophorus maculatus* (Poeciliidae) [16] and are currently known to particularly associate with rDNA and with increased karyotype variability in fishes (e.g., [19–22]).

Although transposable elements are usually silent, bursts of activity and increased copy number can lead to rapid genome diversification between closely related species, as a result of lineage-specific amplification and/or recombination [14]. Due to their high amplification potential, rapid genome expansions are thought to be mediated by transposon activity, especially under conditions that may disrupt normal operation of transposon control systems, like inter-specific hybridization [15]. In fact, hybridization is known to possibly induce transposon activation triggering genome-wide reorganization (genetic and epigenetic) or strongly modifying recombination patterns [12, 23–25]. As a result, gross incompatibilities between species may arise, potentially constituting a first step towards reproductive isolation [14].

To understand the role of repetitive DNAs in the genome differentiation of Iberian Leuciscinae, a molecular cytogenetic survey was conducted in species of the ‘alburnine’, chondrostomine and *Squalius* lineages (see [1]), namely: *Anaecypris hispanica* (AHI), *Achondrostoma oligolepis* (AOL), *Iberochondrostoma lemmingii* (ILE), *I. lusitanicum* (ILU), *Pseudochondrostoma duriense* (PDU), *P. polylepis* (PPO), *Squalius pyrenaicus* (SPY) and natural hybrids of the type *Achondrostoma oligolepis* x *P. polylepis* and *A. oligolepis* x *P. duriense* (designated as AOL x PPO and AOL x PDU hybrids, respectively) (Table 1). They were chosen as representatives of the main Iberian Leuciscinae genera and natural hybrids occurring in Portugal [3, 4]. This is the first report directed at retroelement physical mapping in Cyprinidae that may contribute to the understanding of whether retrotransposons might be at the basis of genome rearrangements, karyotype differentiation or even speciation. The main goals of the present study were: (1) to map the chromosomal distribution and characterize the retroelement Rex3 in these species, (2) to explore the possible transposition (re)activation in the hybrids, and (3) to delineate its association with the translocation of 45S rDNA sites previously identified in such hybrids [11].

**Table 1:** Information regarding the number, sex and location of specimens analysed

Taxa	ID code	Basin	River (Portugal)	Date of collection	No. and sex^a^ of individuals	GenBank
*Anaecypris hispanica*	AHI203, AHI323	Guadiana	Vascão	1999	1 ♂, 1 n.d.	KP001555
*Iberochondrostoma lemmingii*	CGD29	Ardila	Ardila	2011	1 ♀	KP001556
*Achondrostoma oligolepis*	AOL775	Tejo	Nabão	1994	1 ♀	-
*Achondrostoma oligolepis*	CV69	Vouga	Sul	2008	1 n.d.	KJ145023
*Iberochondrostoma lusitanicum*	TR8, TR9	Tejo	Raia	2005	1 ♂, 1 ♀	KP001560
*Pseudochondrostoma duriense*	CTM8, CTM11	Douro	Tâmega	2008	1 ♂, 1 ♀	KP001561
*Pseudochondrostoma polylepis*	ZD62	Mondego	Ceira	2007	1 ♂	-
*Pseudochondrostoma polylepis*	PPO002	Mondego	Mortágua	2007	n.d.	KP001562
*Pseudochondrostoma willkommii*	CGD16	Guadiana	Chança	2011	1 ♀	KP001563
*Squalius pyrenaicus*	SPY207	Guadiana	Vascão	1999	1 ♂	KJ145024
*Squalius pyrenaicus*	MPZ20	Oeste	Cheleiros	2013	1 ♀	-
*Squalius pyrenaicus*	MR305	Tejo	Ocreza	2011	n.d.	-
hybrids *A. oligolepis *x *P. duriense*	CS3, CS20	Douro	Sousa	2008	1 ♂, 1 ♀	KP001557-8
hybrid *A. oligolepis* x *P. polylepis*	CV39	Vouga	Serra	2008	1 ♂	KP001559
hybrid *A. oligolepis* x *P. polylepis*	ZD61	Mondego	Ceira	2007	1 ♂	-
hybrid *A. oligolepis x P. polylepis*	ZD20	Mondego	Mortágua	2007	1 ♀	-

a ♂ = male, ♀ = female, n.d. = not determined

## Results

### Characterization of the Rex3 fragment

**Fig. 1:**
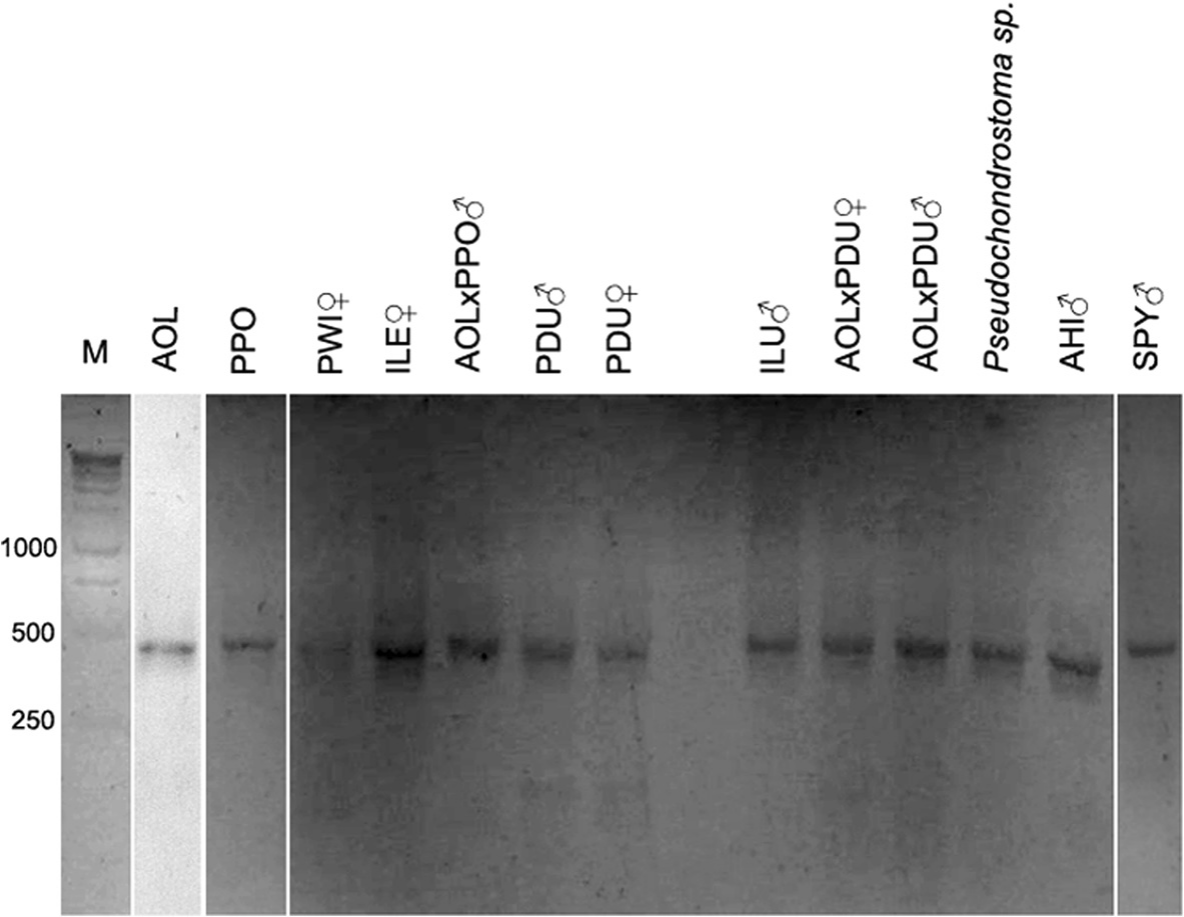
PCR-amplified Rex3 fragment (~460 bp) in Leuciscinae species. M = molecular weight marker (bp), AHI = *Anaecypris hispanica*, AOL = *Achondrostoma oligolepis*, ILE = *Iberochondrostoma lemmingii*, ILU = *I. lusitanicum*, PDU = *Pseudochondrostoma duriense*, PPO = *P. polylepis*, PWI = *P. willkommii*, SPY = *Squalius pyrenaicus*, and AOLxPDU or AOLxPPO = natural hybrids. ♂ = male, ♀ = female

**Fig. 2:**
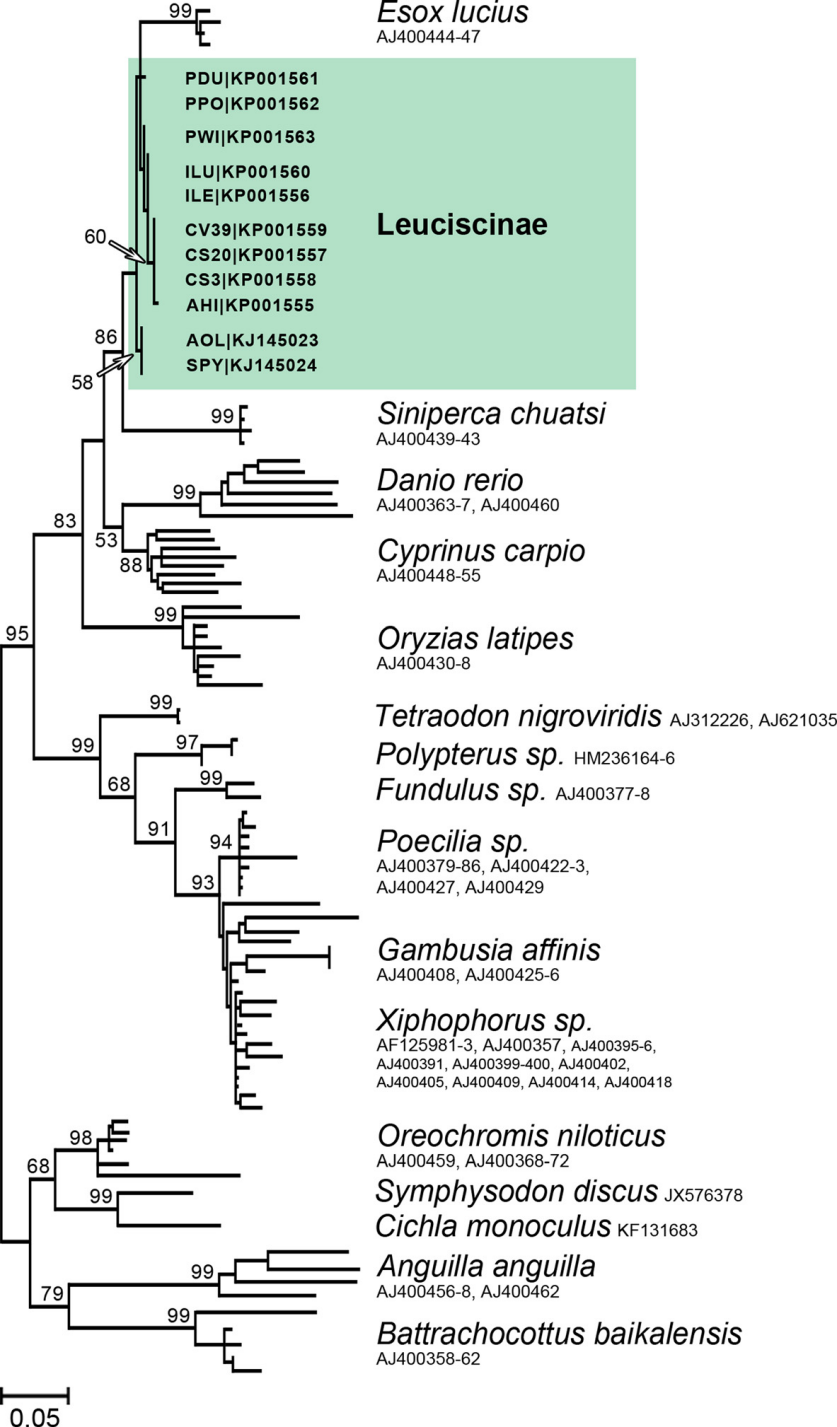
Cladogram of Rex3 partial sequences (maximum-likelihood analysis, bootstrap with 10,000 replicates, Tamura-Nei model, all-sites, very strong) [37, 38]. Only bootstrap values above 50 are shown. GenBank accession numbers are indicated

**Fig. 3:**
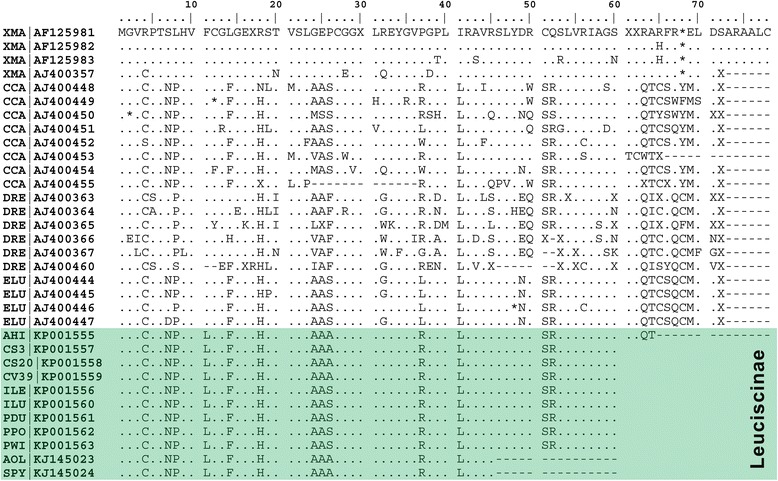
Amino acid sequence alignment of a Rex3 ORF from Iberian Leuciscinae in comparison to *Xiphophorus maculatus* (XMA), *Cyprinus carpio* (CCA), *Danio rerio* (DRE) and *Esox lucius* (ELU). GenBank accession numbers are also indicated. Dots denote similarity, asterisks indicate stop codons and dashes represent gaps

Using the selected pair of Rex3 primers we amplified a single fragment of approximately 460 base pairs (bp) with no significant size variation between species (Fig. 1). Sequencing yielded high quality data for fragments ranging from 326 bp to 468 bp. Sequence homology and genetic distance analyses (Additional file 1) disclosed high sequence similarity within leuciscine sequences (≥98 %). BLASTn megablast analyses confirmed high homology to partial sequences of Rex3 retroelement which were described in the fish families Polypteridae (84–86 %), Cyprinidae (84–91 %), Esocidae (95 %), Adrianichthyidae (84 %), Fundulidae (83 %), Percicthyidae (89 %), Cichlidae (80–86 %), and Tetraodontidae (88 %) (Additional file 1, Fig. 2 and Additional file 2). Phylogenetic analysis of Rex3 sequences did not support the species phylogenetic relationship [1, 26] clustering with the invasive species *Esox lucius* (Fig. 2). Leuciscine Rex3 partial sequences were intact in comparison with those first described in *Xiphophorus maculatus* [16, 18] as the majority of mutations found (~99.9 %) were missense (i.e., coding for a different amino acid) and non-disruptive of the open reading frame (ORF) (Fig. 3). On the other hand, Rex3 sequences of the closest related *Cyprinus carpio* or *Danio rerio* evidenced several substitutions, deletions and stop codons disrupting the same ORF (Fig. 3). Nucleotide substitution rates were overall low (<20 %) with transitions being more common than transversions (Ts/Tv = 1.97).

### Chromosomal distribution of Rex3 retroelement

**Fig. 4:**
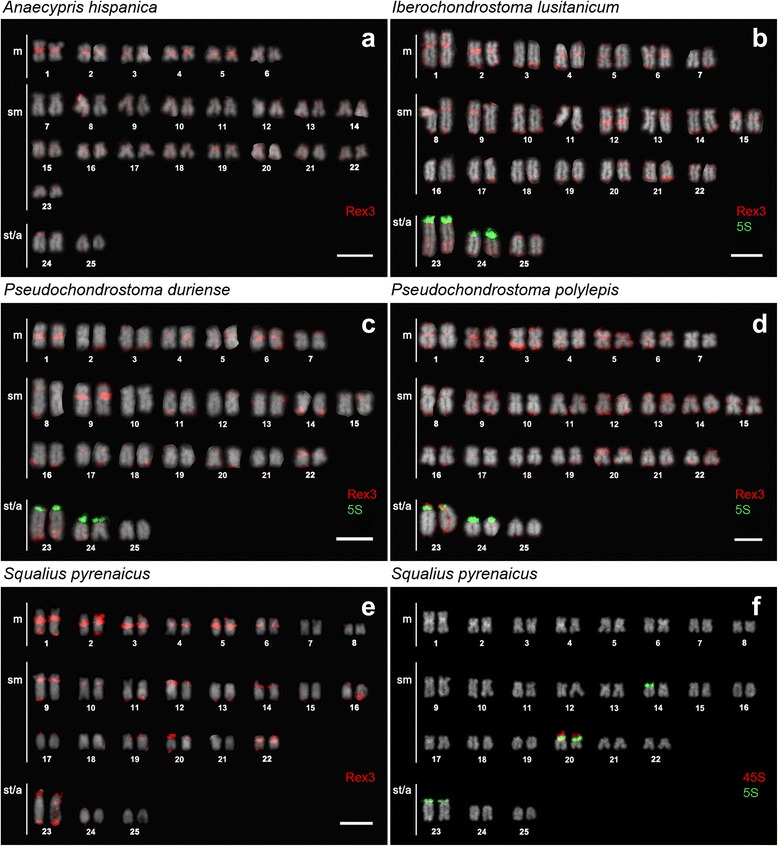
Karyotypes of Iberian Leuciscinae (2n = 50) representative of (**a**) ‘alburnine’, (**b**-**d**) chondrostomine and (**e**-**f**) *Squalius* lineages [1] arranged from chromosomes after FISH with Rex3 fragment (*red*), 5S (*green*) and 45S (*red*) rDNA. Bar = 5 μm

**Fig. 5:**
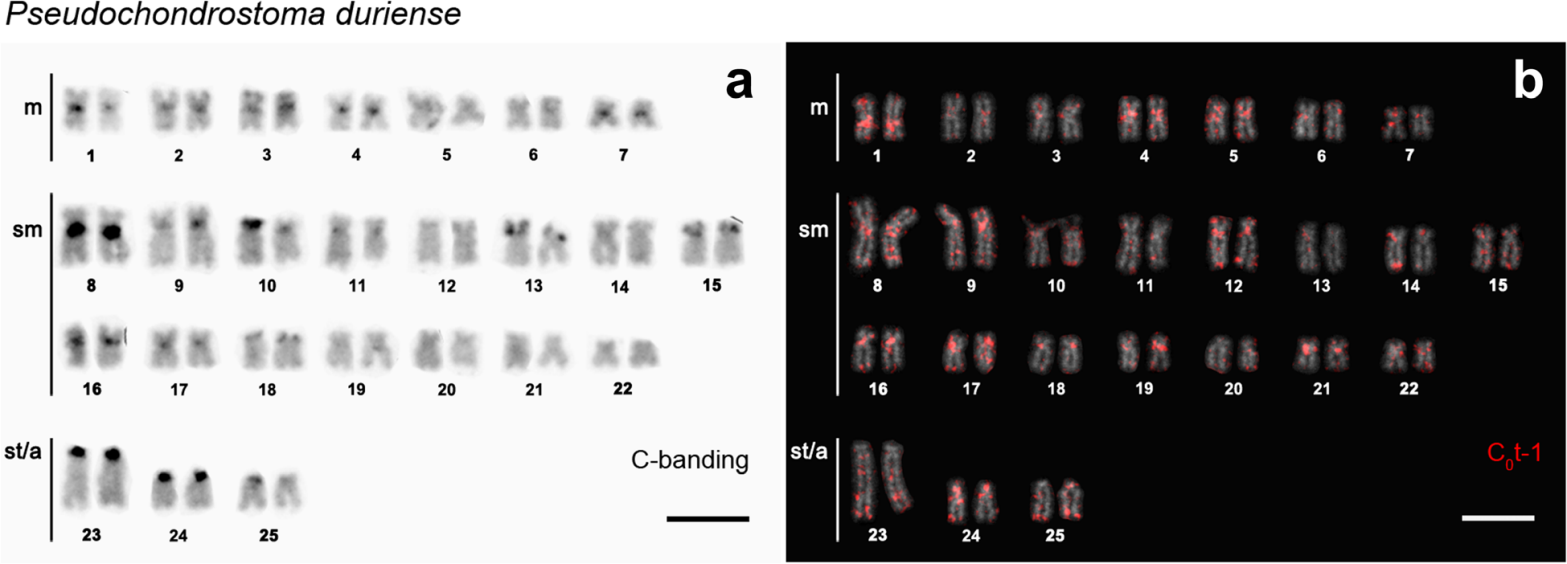
Karyotypes of *Pseudochondrostoma duriense* arranged from chromosomes after (**a**) C-banding with DAPI counterstaining (negative image) and (**b**) FISH with C_0_t-1 DNA fraction (*red*). Bar = 5 μm

All genomes examined for Rex3 distribution evidenced a pattern of larger accumulation on pericentromeric regions and moderately at subtelomeric blocks (Figs. 4a-e). Co-localization with 5S rDNA loci was observed but not with 45S rDNA unless syntenic with 5S rDNA (Fig. 4f; see also [8]), grossly correlating with blocks of constitutive heterochromatin (Fig. 5a) and C_0_t-1 DNA fraction (Fig. 5b). Rex3 clusters were particularly evident in at least 10 chromosome pairs, a pattern that appeared to be shared between the different species under study (Fig. 4). Although less prominent, Rex3 also seemed to be fairly accumulated in the distal part of the 1st pair of subtelo-acrocentric chromosomes of all chromosome sets. Few additional distinctive patterns could be recognized in a species-specific manner; particularly, a big interstitial block in the long arm of chromosome pair No. 12 of ILU (Fig. 4b), two clusters in the short arm of chromosome pair No. 15 of PDU (Fig. 4c), a big telomeric block in chromosome pair No. 3 of PPO (Fig. 4d), and a big pericentromeric block in chromosome pair No. 12 of SPY (Fig. 4e). Conversely, these bands did not correlate to constitutive heterochromatin blocks (not shown) except for PDU (Fig. 5a).

**Fig. 6:**
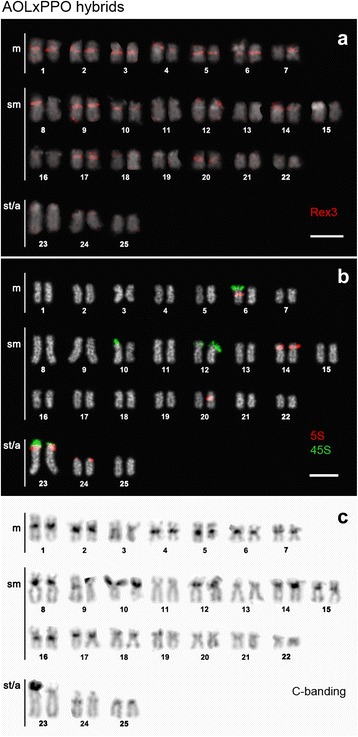
Karyotypes of natural chondrostomine hybrids (2n = 50) of the type AOLxPPO arranged from chromosomes after (**a**) FISH with Rex3 fragment (*red*), (**b**) dual-colour FISH with 5S (*red*) and 45S (*green*) rDNA probes, and (**c**) C-banding with PI counterstaining (negative image). Bar = 5 μm

In the genomes of natural hybrids, Rex3 distribution appeared to agree with the overall pericentromeric/subtelomeric pattern of accumulation already described (Fig. 6a), also correlating with 5S rDNA (Fig. 6b) and constitutive heterochromatin (Fig. 6c). However, differences could be found relative to the parental species: (1) more independent clusters were evident (at least 15 pairs) occurring in all metacentric and most of the submetacentric chromosome pairs (Fig. 6a); and (2) conspicuous bands mapped to the short arms of chromosome pairs Nos. 6, 10 and 12, co-localizing with 45S rDNA clusters as well (Fig. 6a-b).

Interestingly, the pattern of extra or more pronounced bands of Rex3 (Figs. 4b, 6a) or other repetitive sequences (Fig. 5b) present in one of the homologues of the 1^st^ submetacentric chromosome pair was only observed in male specimens of ILU, PDU and one AOLxPPO hybrid.

## Discussion

### Rex3 partial sequence

The retrotransposon Rex3 was found widespread in the genomes of the three Leuciscinae lineages inspected [1] with low (≤2 %) and likely recent sequence divergence. Interestingly, genetic analyses demonstrated higher homology to *Esox lucius* (95 %; Esocidae), i.e., basal Euteleostei, than to *Cyprinus carpio* (>89 %) or *Danio rerio* (>84 %) (Cyprinidae), i.e., basal Otocephala, to which they are more related. The remaining high levels of sequence homology found among the different fish families (80–95 %) strongly suggest sequence conservation despite their distant phylogenetic interrelationships and relatively recent activity. Such discrepancy between Rex3 phylogeny and current fish phylogenies was witnessed before by Volff et al. [18] who proposed several possible explanations. The most adequate seem to be differences in the evolutionary rates between Rex3 sequence and the host genome, since mobile elements multiply independently within the genome; and/or the operation of multiple mechanisms during Rex3 evolution in fish genomes. Nonetheless, present results put forward little sequence variance since divergence of the *Esox* lineage (at least Late Cretaceous), indirectly pointing to either some sort of positive selection to protect Rex3 activity [18]; the existence of a robust mechanism of silencing/regulation of Rex3 activity in the genomes of Leuciscinae preventing its transposition and consequently its differentiation; or possibly a combination of both. Alternatively, all facts point to the possibility of recent horizontal transfer events. This mechanism has already been advanced for other fish species (e.g., [21]), likely via a mutual parasite or through large-scale predation by *E. lucius*, which may have increased its exposure to infection by transposable elements.

The selected pair of primers match some of the reverse-transcriptase domain-encoding regions [16]. All the amplified fragments showed overall low nucleotide substitution rates (<20 %) and intact ORFs with only missense mutations that may result in a slightly different yet functional protein. This result suggests that Rex3 may in fact be active in these genomes in contrast to the highly mutated sequences of *C. carpio* or *D. rerio*. The accumulation of mutations is usually associated with TE senescence [21] which seems to be the case of *C. carpio* and *D. rerio* also proving that the repression mechanisms may differ among even closely related hosts [21].

### Conserved Rex3 distribution in natural populations

The taxa analysed in this study revealed the typical high level of karyotype similarities of Leuciscinae. Rex3 distribution was abundant and compartmentalized in all genomes proving once again widespread and conserved in these lineages. Comparative analysis pointed out possible chromosomal homologies between these long diverged species, probably corresponding to the ancestral condition to all these genera. Assuming the model of vertical transfer, Rex3 genome invasion most certainly preceded their divergence, since it was found quite abundantly even in basal species such as AHI, SPY and ILU (Iberian ‘alburnines’ are thought to have diverged from European Leuciscinae at ca. 12.1 Mya, while Iberian *Squalius* and chondrostomines are believed to have originated around 14.6 Mya and 9.4 Mya, respectively [1]).

Rex retrotransposons have been described and mapped in the teleost orders Characiformes (e.g., [19]), Salmoniformes [22], Perciformes (e.g., [20]), demonstrating various patterns of genomic distribution from dispersed to clustered. In cyprinids, Rex sequences have only been described in the common carp *Cyprinus carpio*, the zebrafish *Danio rerio* [16] and the common bleak *Alburnus alburnus*, with a strong association to the giant B chromosomes found in the latter; but until now there has been no study targeting the physical mapping of such genetic elements to cyprinid genomes. Usually, eukaryotic transposable elements are not randomly distributed along the chromosomes, especially valid for small genomes like those of evolutionary diploid cyprinid fishes [26]; by accumulating within heterochromatin the impact of its presence or activity on the host genome is reduced, while evading negative selection and allowing for their compartmentalization as observed.

Recent studies have further demonstrated linkage of Rex3 with other classes of repetitive DNA such as rDNAs, usually accompanying increased karyotype diversity (e.g., [19–22]). According to Zhang et al. [27], rDNA regions are perfect places for the long-term persistence of transposable elements. In the present investigation this association was clear with 5S rDNA regions but apparently absent from 45S-bearing chromosomes, except when syntenic with 5S rDNA. This association may add up to the presumed flexibility and high variability previously reported [e.g., 8–9, 11], suggesting that transposable elements may be responsible for the multiplication and dispersion of 5S rDNA sites in Leuciscinae as well.

Volff et al. [18] described Rex3 as the most widespread fish retrotransposon with its presence going back as far as 150–200 Mya, despite the discontinued distribution. In this work Rex3 was found fairly distributed at the distal part of the largest subtelo-acrocentric chromosome pair, once again co-localizing with heterochromatin and most likely intercalating with other repetitive sequences. In their work with a WCP (whole chromosome paint probe) specific for this chromosome, Ráb et al. [7] proposed this as the subfamily marker chromosome; likely homologous across this cyprinid lineage and that at least the distal part would be phylogenetically conserved. Accordingly, Rex3 accumulation in this particular region is expected to reflect the same evolutionary history, thus pre-dating the divergence of Leuciscinae subfamily.

Non-heterochromatic species-specific patterns of Rex3 accumulation prove that, even with probable mechanisms of expression regulation, somewhere along the evolution of Iberian species, Rex3 sequences had the opportunity to transpose and accumulate outside the ‘comfort areas’ of heterochromatin shelter. This is also indicative of independent and rapid divergence of species-specific clusters. Mobile elements, as other classes of repetitive sequences, have been demonstrated to accumulate within the sex chromosomes (e.g., [19, 28, 29]). Up to date, no sex-related chromosomes have been convincingly identified or characterized in Leuciscinae but female heterogamety has been proposed for an Iberian *Squalius* species pointing the 1^st^ pair of submetacentric chromosomes as the possible sex elements [30]. Present results, revealed a differential accumulation of Rex3 in that same pair of chromosomes but only in male chondrostomine specimens instead. And even if not associated with evident size polymorphism, such distinction usually represents the early stages of sex chromosome differentiation [19]. However, due to low sample size this correlation must be further validated.

### Rex3 expansion in natural homoploid hybrids

The similar patterns of Rex3 distribution in the inspected leuciscine genomes allow for inferences to be withdrawn for their natural hybrids. The increased number of Rex3-bearing chromosomes suggests an apparent proliferation of Rex3 transposition in the hybrids, now occurring in most of the bi-armed elements of the chromosomal set.

The particular specimen represented in Fig. 6a-b evidenced three translocated clusters of 45S rDNA into chromosomes already bearing 5S rDNA regions (see also [11]). As previously demonstrated for the parental species, Rex3 association with 5S rDNA was retained in the hybrids. But even in 45S rDNA-bearing chromosomes thought to be inherited as a whole (i.e., chromosome pairs No. 10 and 12), a new cluster of Rex3 co-localizing with the 45S rDNA appears as a possible signature of translocation. In light of that, the same may be extended to the newly detected clusters of Rex3 (e.g., chromosome pairs No. 2–4 and 7; Fig. 6a) and to the few differences between homologue pairs (e.g., chromosome pairs No. 4 and 7; Fig. 6a) as a result of conceivable rearrangements as anticipated by Pereira et al. [11]. Similar to recent demonstrations of stress-activated retrotransposons associated with extensive rDNA multiplication ([11, 23] and references therein), hybridization-activated transposition and genome rearrangements are more and more expected to occur in these genomes even if we are not currently able to fully examine them. The increasing number of sequencing data (including other fish species) will soon allow to generate more information on this subject.

## Conclusions

Transposable elements are considered a dynamic force in gene regulation and neo-functionalization, chromosome rearrangements, genome evolution, and even speciation (e.g., [13–15]). By increasing genetic variability, transposable elements promote the evolvability of genomes and species when external conditions change [14]. Therefore, extending the study of these repetitive sequences to other populations and other Leuciscinae representatives will allow to better appreciate karyotype differentiation in the subfamily. Also, the inclusion of more hybrid forms (both homoploid and polyploid) and the follow up of ongoing work on *Squalius sp.* transcriptomics [31] would unquestionably benefit the understanding of transposon distribution, regulation and (re)activation in a scenario of genomic, transcriptomic and epigenetic shock subsequent to the hybridization process. The extensive diversity here again witnessed for Iberian Leuciscinae makes them excellent candidates to explore the processes and mechanisms behind the great plasticity distinguishing vertebrate genomes.

## Methods

### Specimens

Representatives of Iberian Leuciscinae and some of their natural hybrids were selected from the fish/tissue collection of Laboratório de Citogenética, FCUL, Lisbon (Portugal) for cytogenetics and/or molecular analyses. Data on all specimens used in this study were summarized in Table 1.

### Cytogenetics

Chromosome preparations were available from a small bank stored throughout the many years of fish cytogenetic surveys at our lab (see [8]), either obtained from *in vivo* kidney preparations or from fin fibroblast cultures. Genomic DNA was extracted from fin clips or muscle by isopropanol/ethanol precipitation and the set of specific FISH probes included: (1) the DNA fraction enriched for repetitive sequences – C_0_t-1 DNA [28], (2) the PCR-amplified 5S rDNA gene, (3) a clone containing the 45S rDNA sequence [8], and (4) a PCR-amplified Rex3 fragment using the pair of primers F3 and R3 originally designed by Volff et al. [16]. All sequences were labelled with Digoxigenin or Biotin by nick translation (Roche), dissolved in hybmix (50 % deionised ultrapure formamide, 10 % dextran sulphate, 2x SSC, pH 7.0) to a final concentration of 20 ng.uL^−1^ and mapped in the chromosome sets of the species analysed. All chromosome preparations were equally treated except for the denaturation step (67 °C in 70 % formamide, 2x SSC, pH 7.0) which was longer for the material obtained using *in vivo* (3 min.) than *in vitro* procedures or in older preparations (1 min). Probes were denatured for 10 min at 75 °C and hybridizations proceeded overnight at 37 °C in a humidified chamber. C-banding followed Sumner [32] with DAPI or PI counterstaining. Images (Olympus, Japan) were processed as a whole using pseudo-colouring, over-layering and brightness/contrast tools (Adobe Photoshop CS5). Karyotype assembly followed Levan et al. [33].

### Sequence analysis

Before using it as a probe for FISH procedures, the identity of the Rex3 fragment was confirmed by sequencing and BLASTn analysis [34]. The purified fragment was cloned into pDrive Cloning Vector (Qiagen) and transformed into EZ Competent Cells (Qiagen) for long time storage/access and sequencing (STAB Vida, Portugal). Sequences were edited and aligned using ClustalW [35] and subjected to a megablast analysis to retrieve highly similar sequences deposited in GenBank database [36]. ORFs were predicted using the ORF finder tool and amino acid sequences were deduced from nucleotide sequences using BioEdit [35]. From the 96 annotated Rex3 sequences to date (25/03/2015) used to build the Rex3 cladogram (Fig. 2), 31 were randomly selected (one representative per species) to estimate the evolutionary divergence (Additional file 1) based on the number of base substitutions per site and the Kimura 2-parameter model [37, 38]. All ambiguous positions were removed for each sequence pair resulting in a total of 3360 positions in the final dataset. The patterns of nucleotide substitution were estimated via Maximum Likelihood Composite [37] for the 96 Rex3 sequences. Codon positions included 1st + 2nd + 3rd + Noncoding and all ambiguous positions were removed for each sequence pair resulting in a total of 421 positions in the final dataset. All sequences were deposited in GenBank (Table 1).

## Ethics statement

All procedures were performed in compliance with ASAB/ABS guidelines.

## Additional files

Additional file 1:
**Estimates of evolutionary divergence between selected Rex3 partial sequences (Neighbour-Joining method, 1000 replicates) [** 37, 38 **].** Species are referred to by a three-letter code followed by GenBank accession numbers (alphabetical order). For more details refer to the text and Fig. 2. (XLS 42 kb) Additional file 2:
**BLASTn megablast results (limit set: 1000 hits).** Minimum, maximum (green) and calculated average (grey) percentage of similarity for each pair of species as well as the overall average (yellow) are shown. Fish systematics followed. (XLSX 16 kb)

## Abbreviations

DAPI: 4,6-diamidino-2-phenylindole
Mya: Million years ago
ORF: Open reading frame
PI: Propidium iodide
rDNA: Ribosomal DNA
SSC: Saline sodium citrate
WCP: Whole chromosome paint probe
